# Letter to Dr. Jenner, on Vaccine Inoculation

**Published:** 1802-05

**Authors:** Tho. Bayley

**Affiliations:** in the Chair. Golden Lion, Ipswich


### *To the Editors of the Medical and Phyfical Journal.*

Gentlemen,

I Have to requeft that you will give in your next Journal a place to the above letter to Doctor Jenner, which was unanimoufly agreed to at a meeting of the Suffolk Society of Surgeons, held this day.

*Golden Lion, Ipfwich,* I am, &c.

*April, 19, 1802.* THO. BAYLEY, in the Chair.

---

### To Dr. JENNER.

Sir,

WE the underfigned Members of the Suffolk Society of Surgeons, having had ample opportunity of experiencing the fafety and efficacy of the Vaccine Inoculation, in preventing Small Pox, introduced, and now happily brought into general ufe, by your Difcovery, founded upon accurate and patiently conducted experiments, think it our duty to congratulate you, and to return you our grateful thanks for the invaluable advantages that the community at large have derived from your labours; which, in the courfe of a few years, promife entirely to eradicate the dangerous and loathfome difeafe of Small Pox.

We have the honor to be, Sir,
Your moft obedient and moft humble fervants,

*Golden Lion, Ipfwich,* Edward B. Beck, Prefident.

*April 19, 1802.* William Spack, Vice-Prefident.

| | |
|---|---|
| Nathaniel Bucke, | Jn. Morgan |
| Thomas Bayley, | Robt. Fitch, |
| Robert Abbot, | Richard Freeman |
| William Hamilton, M. D. | George D. Lynn |
| Alexander Bartlet | Nathl. Moore |
| W. Leedes | John Clubbe, M. D. |
| George Stebbing | John Bucke. |

